# When diet meets genetics

**DOI:** 10.7554/eLife.92714

**Published:** 2023-10-19

**Authors:** Karthickeyan Chella Krishnan

**Affiliations:** 1 Department of Pharmacology and Systems Physiology, University of Cincinnati College of Medicine Cincinnati United States

**Keywords:** BXD mice, human, uk biobank, colon, systems genetics, gut health, Mouse

## Abstract

Gene expression profiling of a diverse mouse population helps to decipher how a fat-rich diet contributes to inflammatory bowel disease.

**Related research article** Li X, Morel J-D, Benegiamo G, Poisson J, Bachmann A, Rapin A, Sulc J, Williams E, Perino A, Schoonjans K, Sleiman MB, Auwerx J. 2023. Genetic and dietary modulators of the inflammatory response in the gastro-intestinal tract of the BXD mouse genetic reference population. *eLife* **12**:RP87569. doi: 10.7554/eLife.87569.

Inflammatory bowel disease (IBD) encompasses a group of conditions – including ulcerative colitis and Crohn’s disease – characterized by chronic gut inflammation. Quality of life can be significantly negatively impacted by these conditions, and they can also increase the risk of colorectal cancer. How and why IBD emerges remains poorly understood.

Our digestive system constantly adapts to what we eat, with different foods triggering changes to the way our gut cells express their genes. Factors like diet, genetics and the environment can all play a role in causing gut inflammation, which can become chronic and result in damage (Huang et al., 2017; Enriquez et al., 2022). In particular, the global rise in IBD incidence has been partly linked to increased consumption of fat-rich diets (Maconi et al., 2010; Hou et al., 2011). However, while studies in mice have shown that such diets can increase gut inflammation (Wang et al., 2023), in humans the effects vary among individuals (Zeevi et al., 2015). Understanding how genes and diet interact during early gut inflammation is therefore crucial to understand how IBD starts and to pinpoint the genes involved. It can be difficult to conduct this work due to the wide genetic diversity among humans and the challenges of creating controlled environments to study them in.

Systems genetics is an approach that allows scientists to dissect how various environmental and genetic factors work together to influence disease susceptibility and other traits (Seldin et al., 2019). It relies on ‘libraries’ of mice strains, such as the BXD recombinant inbred family, which have been created to have well-documented genetic differences (Ashbrook et al., 2021). By exposing this ‘genetic reference population’ to various controlled settings, it becomes possible to precisely examine interactions between genes and the environment.

Now, in eLife, Maroun Sleiman, Johan Auwerx of the École Polytechnique Fédérale de Lausanne and colleagues – including Xiaoxu Li as first author – report that a systems genetics approach to studying the relationship between a fat-rich diet and gut inflammation can identify candidate genes that might influence susceptibility to IBD in humans (Li et al., 2023).

First, Li et al. fed 52 BXD mouse strains with either a regular or fat-rich diet. Analyzing the gene expression profiles of the mice guts showed that overall, the fat-rich diet led to increased expression of genes involved in inflammatory pathways. However, much like in humans, the mice strains displayed diverse gene expression profiles. In fact, several strains were resistant to dietary changes, demonstrating that genetic differences can override the effect of diet.

Next, Li et al. compared the gene expression profiles of the BXD mice fed the fat-rich diet with existing datasets from mice and humans with IBD. On average, the genes dysregulated in IBD and in BXD mice were the same, indicating that the fat-rich diet had led to IBD-like gut inflammation. Individually, the gene expression of each strain could be used to classify the strain as as ‘susceptible’, ‘intermediate’, or ‘resistant’ to IBD-like inflammation.

Finally, network modelling approaches were used to group genes that are co-expressed or tend to work together in BXD mice. In animals fed fat-rich diets, some of the resulting ‘modules’ were enriched with genes that are dysregulated in IBD, with two containing genes involved in regulating gut inflammation. Li et al. then used three criteria to identify genes within the modules that might be key to IBD inflammation. Based on the existing human datasets, genetic variants of two of the genes that met these criteria – *Epha6 and Muc4 –* are also associated with ulcerative colitis, suggesting they could be key to regulating gut inflammation (Figure 1).

**Figure 1.**
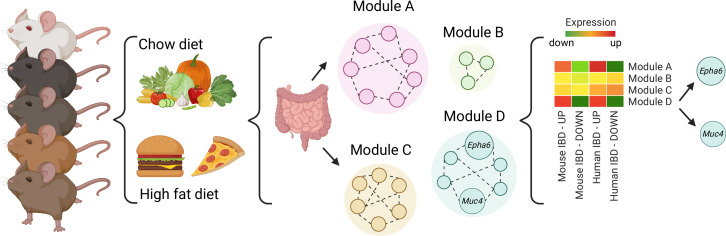
A systems genetics approach to identifying genes involved in diet-related gut inflammation. A diverse group of 52 BXD mouse strains were exposed to regular (chow) or fat-rich diets. Their guts were then collected and gene expression was measured. Further network modeling analyses revealed that the fat-rich diet led to gut inflammation gene expression profiles similar to those in existing, published mouse and human IBD datasets and identified two modules of interest (Modules A and D). Within module D, two genes (*Muc4* and *Epha6*) were identified as candidates that may control gut inflammation in IBD as their genetic variants were also associated with ulcerative colitis in humans. IBD, Inflammatory Bowel Disease.

Image credit: Figure created using BioRender.

The findings, obtained using a powerful combination of systems genetics and pre-published datasets, help to shed light on how genetic makeup and diet dictate vulnerability to IBD. The work also provides a dataset that can be used to generate new ideas for future research, which is important for developing better preventive and treatment strategies for gut-related inflammatory disorders. It also remains to be seen whether the candidate genes identified using this approach can be used to manipulate vulnerability to gut inflammation.
